# Testosterone and cortisol responses to ß‐hydroxy ß‐methylbutryate consumption and exercise: A meta‐analysis

**DOI:** 10.1002/fsn3.2887

**Published:** 2022-04-15

**Authors:** Li Zhao, Mohamad Mohammad

**Affiliations:** ^1^ 118385 College of Physical Education Chengdu University of TCM Chengdu China; ^2^ 48516 Shahid Sadoughi University of Medical Sciences and Health Services Yazd Iran

**Keywords:** cortisol, exercise, HMB, meta‐analysis, testosterone, β‐hydroxy β‐methylbutryate

## Abstract

**Background:**

β‐hydroxy β‐methylbutryate (HMB) is a metabolite of leucine amino acid and it has several ergogenic benefits. Previous studies also showed that it may affect beneficially the testosterone and cortisol concentration in athletes. Due to the contradiction results between studies, we aimed to conduct this meta‐analysis to assess the HMB supplementation effect on testosterone and cortisol in trained athletes.

**Methods:**

Scopus, Medline, and Google scholar were systematically searched up to August 2021. The Cochrane Collaboration tool for evaluating the risk of bias was applied for assessing the studies' quality. Random‐effects model, weighted mean difference (WMD), and 95% confidence interval (CI) were used for estimating the overall effect. Between‐study heterogeneity was evaluated applying the chi‐squared and I^2^ statistic.

**Results:**

Seven articles were included in the meta‐analysis. Although the meta‐analysis generally showed that HMB consumption did not have any effect on the cortisol and testosterone concentration (*p* > .05), subgroup analysis based on the exercise type showed a significant decrease in the cortisol concentration in resistance training exercises (WMD = −3.30; 95% CI: −5.50, −1.10; *p* = .003) and a significant increase in the testosterone concentration in aerobic and anaerobic combined sports (WMD = 1.56; 95% CI: 0.07, 3.05; *p* = .040).

**Conclusion:**

The results indicate that HMB supplementation in athletes can reduce the concentration of cortisol in resistance exercises and increase the concentration of testosterone in aerobic and anaerobic combined exercises. Nevertheless, more studies are required to confirm these results.

## Graphical abstract

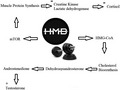

## 1 INTRODUCTION

In recent years, an enormous growth of interest has been placed on nutritional supplements for enhancing training‐induced adaptations. This interest has been motivated by this theory that nutritional supplements may improve performance by increasing anabolic and decreasing catabolic effects, thereby improving body composition and decreasing exercise‐induced muscle damage (Fernández‐Landa et al., 2019; Naderi et al., 2016; Ratamess, 2019).

One potential nutrient is the leucine metabolite β‐hydroxy β‐methylbutryate (HMB) supplementation, that it may result in several ergogenic advantages, including anticatabolic (Knitter et al., 2000), anabolic (Jówko et al., 2001), and lipolytic impacts (Gallagher et al., 2000). HMB, a leucine and 2‐ketoisocaproic acid metabolite, has been suggested to enhance recovery and improve skeletal muscles during high‐volume and high‐intensity exercises (Fernández‐Landa et al., 2020; Portal et al., 2011; Wilson, Fitschen, et al., 2013; Zanchi et al., 2011). It has been reported that HMB increased strength (Nissen et al., 1996), power (Kraemer et al., 2009), and lean body mass (Gallagher et al., 2000; Jówko et al., 2001) through muscle breakdown inhibition during exercise and protein synthesis increase after exercise. Recent data suggested that the advantages of HMB supplementation may be attributed to the some mechanisms including (1) upregulation of IGF‐I (Insulin‐like growth factor 1) expression in skeletal muscle, (2) stimulating synthesis of protein via the mammalian/mechanistic target of rapamycin complex 1 (mTOR), (3) membrane stabilization of muscle cells, and (4) decreasing proteolysis by inhibition of the ubiquitin‐proteasome pathway (J Cruz‐Jentoft & A., 2018; Zanchi et al., 2011).

Previous studies also have found that supplementation with HMB resulted in beneficial effects on performance without increasing anabolic hormones, including IGF‐1, growth hormone, and testosterone and decreasing catabolic stress hormones such as cortisol (Asadi et al., 2017a; Portal et al., 2011a). HMB also may increase androgen concentration through increasing cholesterol, drawing on this hypothesis that, HMB is metabolized to β ‐hydroxy β ‐methylglutaryl CoA (HMG‐CoA) which is a main source of cholesterol synthesis and precursor of androgens such as testosterone (Nissen & Abumrad, 1997).

While several studies have supported the HMB beneficial effect in physical activity and clinical condition, some other studies have shown conflicting results about the HMB supplementation effect on testosterone and cortisol concentration in athletes (Park et al., 2013; Smith et al., 2004; Van Someren et al., 2005). For example, Durkalec‐Michalski et al. reported an increase in testosterone after 12 weeks of supplementation with HMB (3 × 1 g HMB/day) in 42 highly trained males (Durkalec‐Michalski et al., 2017). However, in another study, testosterone and cortisol did not change in 28 male trained athletes after 6 weeks of HMB supplementation (3 g/day) (Crowe et al., 2003).

From our point of view, no systematic review and meta‐analysis has been conducted over the HMB supplementation effect on testosterone and cortisol in trained athletes. Moreover, due to the contradiction results between studies and common HMB consumption as an ergogenic aid, particularly among athletes, we aimed to conduct this systematic review and meta‐analysis to assess the effects of HMB supplementation on testosterone and cortisol in trained athletes.

## 2 METHODS

### 2.1 Search strategy

This systematic review and meta‐analysis was reported according to the guidelines of Preferred Reporting Items for Systematic Reviews and Meta‐Analyses (PRISMA) (Liberati et al., 2009). A computerized search was conducted from inception to August 2021 using various databases including PubMed, ISI Web of Science, Scopus, and supplementary Google Scholar search. The following terms and their combinations of MeSH and non‐MeSH terms were applied, including: “beta hydroxy beta methylbutyrate,” “hydroxy methyl butyrate,” “HMB,” “exercise,” “sport supplements,” “endocrine response,” “cortisol,” and “testosterone.” Reference lists of all studies were checked for further eligible article identification.

### 2.2 Eligibility criteria

Studies were selected by applying the following Population‐Intervention‐Comparator‐Outcomes‐Study design (PICOS) criteria (Liberati et al., 2009): (1) healthy subjects received oral HMB supplementation, as a nutritional strategy before and after exercise; (2) original randomized‐controlled trial researches; and (3) reporting at least one outcome measure of hormones (cortisol and testosterone). Studies were not conducted by using multiple supplementations, (HMB in conjunction with other supplements like creatine (Crowe et al., 2003), arginine, lysine (Flakoll et al., 2004), and adenosine monophosphate (Lowery et al., 2016)). There were restrictions on the performed postexercise and studies that assessed the acute effect of HMB on the hormonal response (Townsend et al., 2015; Wilson, Lowery, et al., 2013).

### 2.3 Selection strategy

After computerized search, titles and received papers' abstracts were screened by the search strategy. Two authors (LG and M.M) selected articles based on the inclusion criteria independently. Articles including eligibility criteria in the title and abstract screening were selected to be checked by full manuscript. Parallel clinical trials were selected by applying a control group or crossover design in the systematic review and meta‐analysis. All classified trials were retrieved by either of the authors. According to the data within the full information, we used a form to select the studies eligible for inclusion in the meta‐analysis. Contradictions between the authors were solved by consensus or third researcher (L.Z).

### 2.4 Data extraction

M.M and LG extracted the following needed information: first author's name, year and publication country, design of studies, gender and age of subjects, HMB dose and sample size, duration of intervention, and training status. Also, we extracted mean and standard deviation (*SD*) of serum hormones' status (cortisol and testosterone) at baseline and after the intervention.

### 2.5 Study quality

Given that trial inclusion with a high risk of bias may distort the outcomes of a meta‐analysis (Higgins et al., 2011; Liberati et al., 2009), the Cochrane Collaboration tool was used for evaluating the risk of bias. The following items were evaluated: randomization sequence generation; allocation concealment; blinding of participants, personnel, investigator, and assessor; attrition rates; and companies' financial interest. These items were given high, unclear, or low risk of bias rating. A study was categorized as having low, medium, or high risk according to the key areas of allocation concealment, reporting of attrition rates, and participants and assessor (high = all 4 factors rated high, medium = 2 or 3 factors rated high or unclear, and low = all key areas rated low).

### 2.6 Analyses and measures of treatment effect

For each trial, mean differences and standard deviation were calculated for continuous variables. The differences of mean changes in cortisol and testosterone concentration and their correspondence *SD* were extracted as the effect size. In trials that the standard error (SE) measure was reported, SE was converted to *SD* applying this formula: *SD*=SE×√*n* (*n*=number of subjects in each group). Between‐study heterogeneity was assessed using the chi‐squared (χ^2^) test and quantified applying the I^2^ statistic, which shows the total variation percentage across studies that is ascribable to heterogeneity rather than to chance. Significant heterogeneity was defined with a *p*‐value of <.05.

The random effects model was applied to calculate the weighted mean differences (WMDs) with 95% confidence intervals (CIs) for estimating the overall effect. To evaluate whether the outcomes could have been altered by a single study distinctly, a sensitivity analysis was performed (Tobias, 1999). Also, subgroup analysis was conducted, based on the sports type (resistance training or aerobic and anaerobic combined training) and duration of studies (over six weeks and 6 weeks or lower). Publication bias was evaluated by Egger's regression asymmetry test and Begg's rank correlation test. Funnel plots also depicted the effect sizes against their corresponding standard errors. Statistical analyses were conducted applying STATA 11.2 software (StataCorp).

### 2.7 NutriGrade

To assess the overall quality of current meta‐analysis with regard to the HMB supplementation on testosterone and cortisol concentration, we used the NutriGrade scoring system (Schwingshackl et al., 2016). NutriGrade uses a scoring system (scored 0 up to 10) to judge the quality of meta‐analyses of RCTs conducted in nutrition science. This tool considers the following criteria: (1) risk of bias (3 points); (2) precision (1 point); (3) heterogeneity (1 point); (4) directness (1 point); (5) publication bias (1 point); and (6) funding bias (1 point) and study design (2 point). In order to evaluate the validity of evidence, NutriGrade suggests 4 categories, including: (1) high (≥8 points); (2) moderate (6–7.99 points); (3) low (4–5.99 points); and (4) very low (≤3.99 points) (Schwingshackl et al., 2016).

## 3 RESULTS

### 3.1 Search outcomes and overview of included studies

**FIGURE 1:**
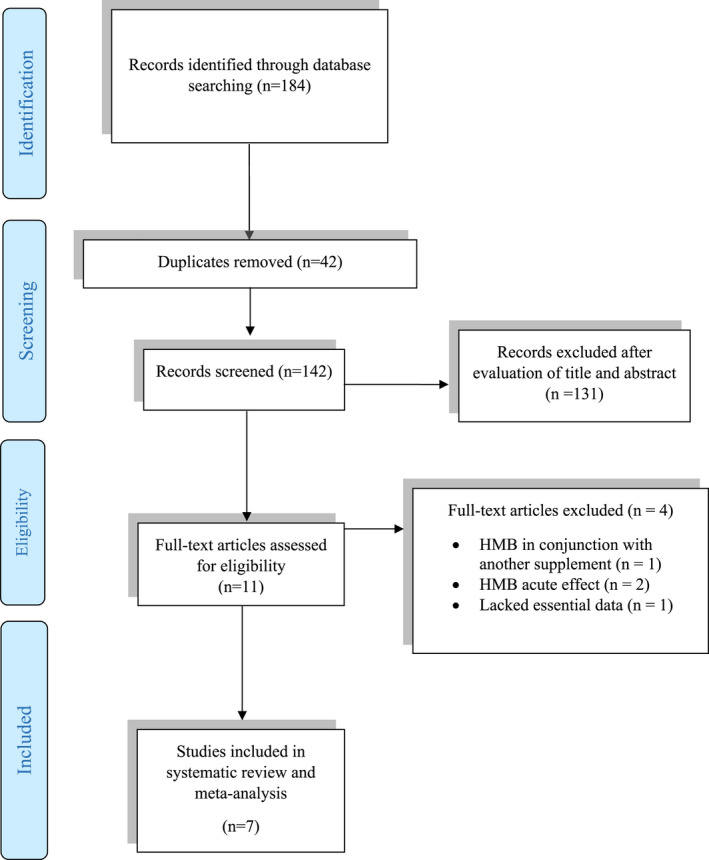
Flow diagram of the study selection process

Our initial search led to 184 relevant studies. After removing duplications, a wide range of screening of the titles and abstracts, a careful assessment was performed on 142 related articles. Of these, 11 articles remained after considering the inclusion and exclusion criteria for eligibility. Eventually, 7 articles, including, 8 effect sizes, which studied a total of 235 participants aged 16.1–24.9 years, were identified in the current systematic review and meta‐analysis. Figure 1 represents the selection process and reasons for excluding the studies. The data in Table 1 illustrate the main characteristics of the articles in our systematic review and meta‐analysis.

**TABLE 1:** Characteristics of the included studies

Author	Study design characteristics					Average age (y)	Sample size		Evaluated hormones
	Design	Country	HMB dose (g)	Duration (w)	Gender		HMB	Control	
Durkalec‐Michalski et al. (2017)	C‐O	Poland	3	12	M	22.8	42	42	C, T
Asadi et al. (2017)	P	Iran	3	6	M	21.4	8	8	C, T
Durkalec‐Michalski and Jeszka (2015)	C‐O	Poland	3	12	M	19.5	16	16	C, T
Portal et al. (2011)	P	Israel	3	7	M & F	16.1	14	14	C, T
Hoffman et al. (2004)	P	USA	3	1.5	M	20.7	15	15	C, T
Crowe et al. (2003)	P	Australia	3	6	M	24.9	11	5	C, T
Slater et al. (2001)	P	Australia	3 (Standard)	6	M	24.6	7	7	C, T
Slater et al. (2001)	P	Australia	3 (Time Releasing)	6	M	24.6	8	7	C, T

Abbreviations: C, Cortisol; C‐O, cross‐over studies; F, Female; g, gram; HMB, β‐hydroxy β‐methylbutyrate; M, men; P, Parallel; T, Testosterone; t, trained; U, untrained; W, weeks; Y, years.

In brief, the studies (by 235 participants) were published between 2001 and 2017. The duration of these RCTs ranged between 1.5 and 12 weeks. Two studies used a randomized crossover design (Durkalec‐Michalski & Jeszka, 2015; Durkalec‐Michalski et al., 2017), one of them was also single blind (Hoffman et al., 2004) and the other studies had the design of double‐blind. Two studies were conducted in Poland (Durkalec‐Michalski & Jeszka, 2015; Durkalec‐Michalski et al., 2017), 1 in the United States (Hoffman et al., 2004), 1 in Iran (Asadi et al., 2017), 2 in Australia (Crowe et al., 2003; Slater et al., 2001), and 1 in Israel (Portal et al., 2011). The effect of exercise on testosterone and cortisol concentration was examined in 7 studies (Asadi et al., 2017; Crowe et al., 2003; Durkalec‐Michalski & Jeszka, 2015; Durkalec‐Michalski et al., 2017; Hoffman et al., 2004; Portal et al., 2011; Slater et al., 2001); and 8 effect sizes were extracted from these studies for testosterone and cortisol concentration. The total number of subjects who completed the studies with inclusion criteria was 121 in the intervention and 107 in the placebo groups. The dose of HMB supplementation was 3 g/day in all studies.

### 3.2 Risk of bias

The quality of the studies is presented in Table 2. Briefly, random sequencing generation was unclear in 3 studies (Crowe et al., 2003; Hoffman et al., 2004). Participants and outcome blinding and incomplete outcome data were high and unclear risk of bias in only two studies, respectively (Crowe et al., 2003; Slater et al., 2001). And then ranked as low risk of bias for 5 studies (Asadi et al., 2017; Durkalec‐Michalski & Jeszka, 2015; Durkalec‐Michalski et al., 2017; Hoffman et al., 2004; Portal et al., 2011), medium risk of bias for 1 study (Slater et al., 2001), and high risk of bias for 1 study (Crowe et al., 2003). Most of studies had a low risk of bias for selective reporting and other sources of bias except for one study (Crowe et al., 2003).

**TABLE 2:** Cochrane risk of bias assessment

Study	Random Sequence Generation	Allocation concealment	Blinding of participants and personnel	Blinding of outcome assessment	Incomplete outcome data	Selective outcome reporting	Other sources of bias	Overall risk of bias
Durkalec‐Michalski et al. (2017)	L	L	L	L	L	L	L	L
Asadi et al. (2017)	L	U	L	L	L	L	L	L
Durkalec‐Michalski and Jeszka (2015)	U	L	L	L	L	L	L	L
Portal et al. (2011)	L	L	L	L	L	L	U	L
Hoffman et al. (2004)	U	U	L	L	L	L	L	L
Crowe et al. (2003)	U	U	H	U	H	L	L	M
Slater et al. (2001)	U	L	U	U	U	L	L	M

Abbreviations: H, high risk of bias; L, low risk of bias; M, medium risk of bias; U, unclear risk of bias.

### 3.3 Findings from the meta‐analysis

#### 3.3.1 Effects of HMB supplementation on serum cortisol concentration

**FIGURE 2:**
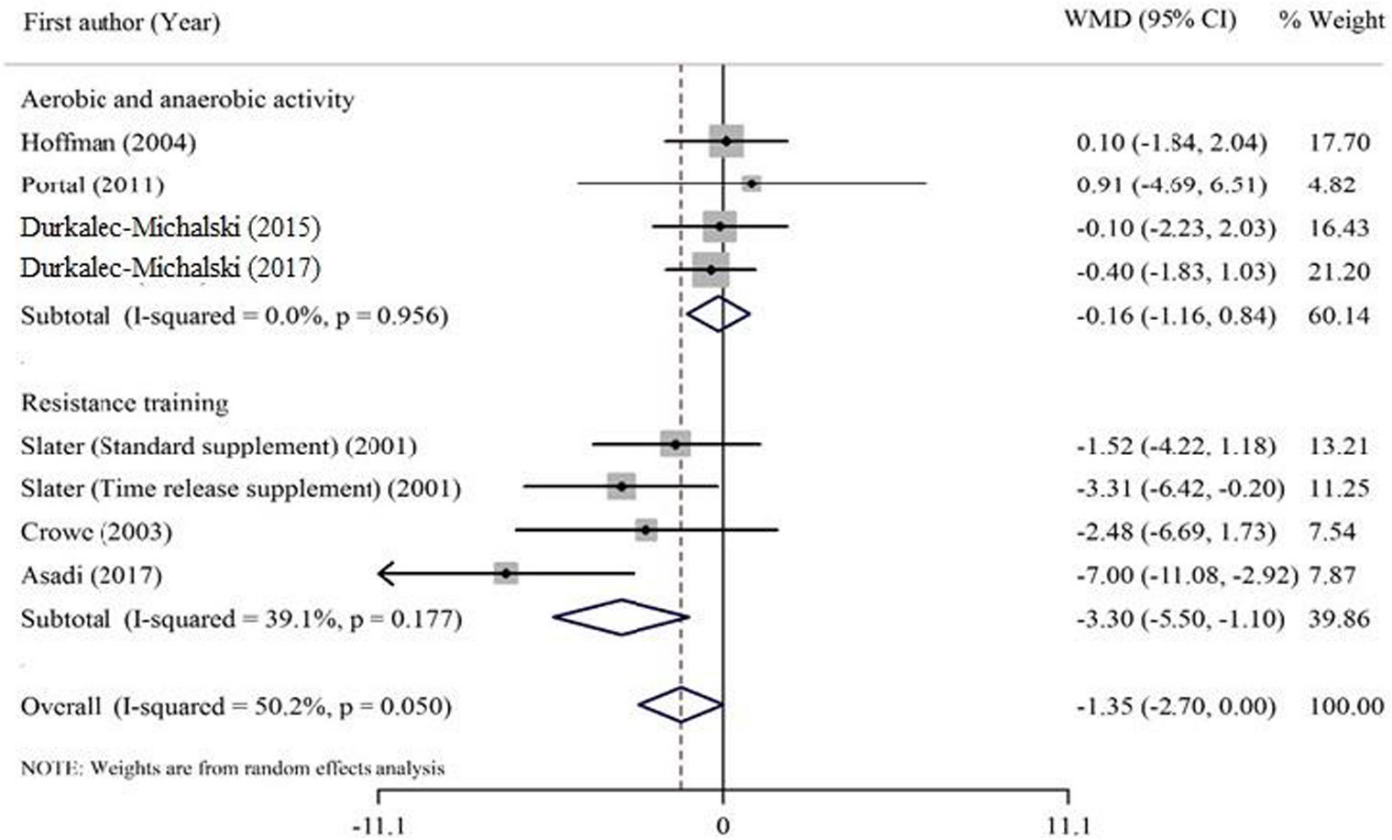
Forest plot representing the effect of HMB supplementation on the cortisol concentration. The analysis was done using a random effects model, subgroup analysis was performed based on the type of exercise activities

Overall, HMB consumption had no significant effect on cortisol concentration. There was no significant heterogeneity among the studies. Subgroup analyses of different types of exercise showed that cortisol concentration significantly reduced after resistance exercises, whereas it had no significant effect on cortisol concentration in aerobic and anaerobic combined activities. The results of subgroup analysis are shown in Table 3 and the forest plot is shown in Figure 2.

**TABLE 3:** Effect of HMB supplementation on cortisol concentration in athletes, subgroup analyses based on study duration and type of exercise activities (all analyses were conducted using random effects model)

Study group	No. of effect sizes	Meta‐analysis		Heterogeneity			
		Effect Mean difference (95%CI)	*P* effect	*Q* statistic	*P* within group	*I^2^* (%)	*P* between group
Duration							
>6 weeks	3	−0.25 (−1.42, 0.90)	0.668	0.23	0.893	0.00	0.144
≤6 weeks	5	−2.47 (−4.72, −0.21)	0.032^*^	10.96	0.027	63.50	
Type of exercise activities							
Aerobic and anaerobic combined	4	−0.16 (−1.16, 0.84)	0.753	0.32	0.956	0.00	0.003^*^
Resistance training	4	−3.30 (−5.50, −1.10)	0.003^*^	4.93	0.177	39.1	
Overall	8	−1.35 (−2.70, 0.002)	0.050	14.05	0.050	50.20	‐

Bold values provide significancy *p < .05.

#### 3.3.2 Effects of HMB supplementation on testosterone concentration

**FIGURE 3:**
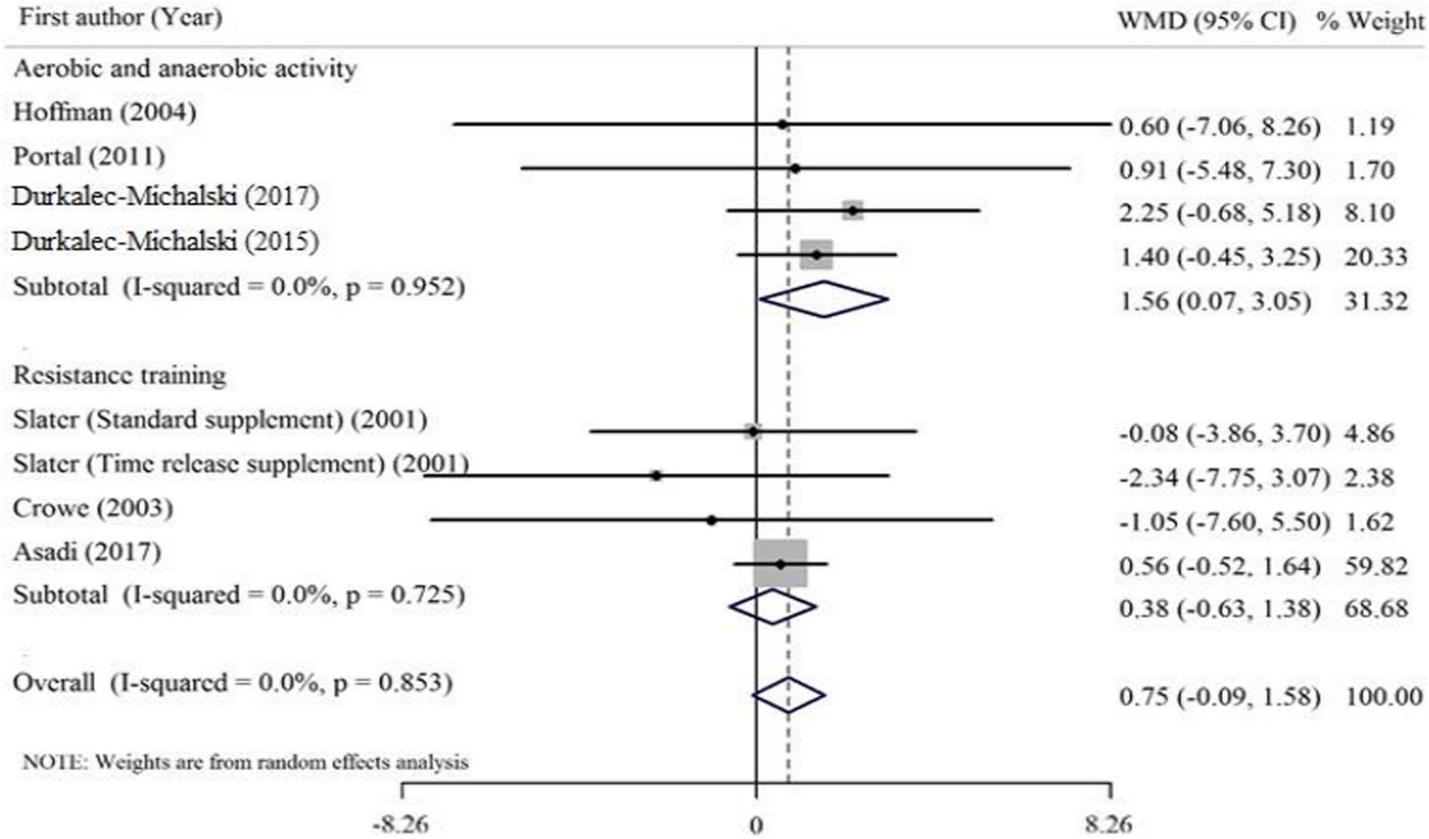
Forest plot representing the effect of HMB supplementation on the testosterone concentration. The analysis was done using a random effects model, subgroup analysis was performed based on the type of exercise activities

**FIGURE 4:**
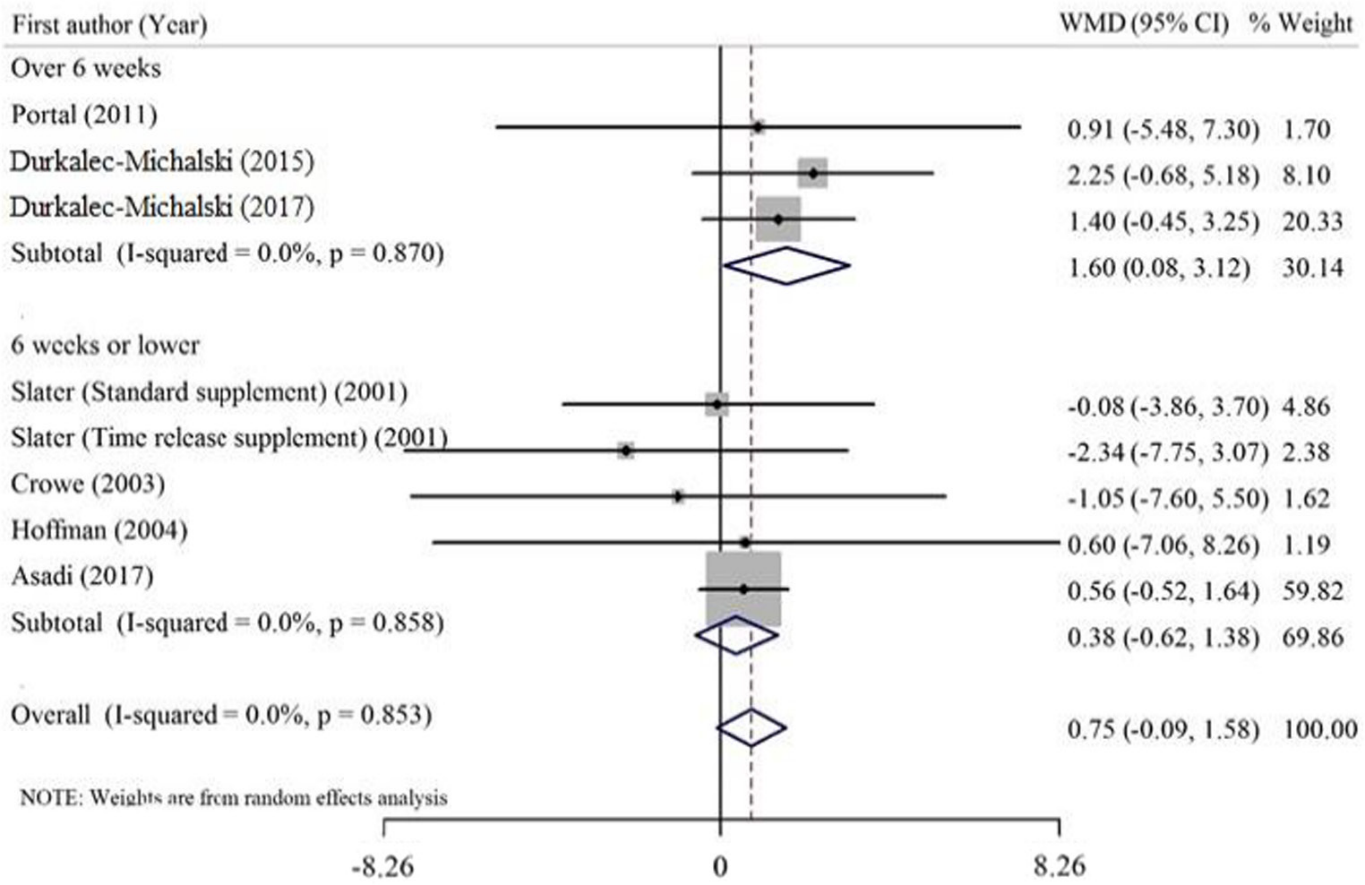
Forest plot representing the effect of HMB supplementation on the testosterone concentration. The analysis was done using a random effects model, subgroup analysis was performed based on the duration of the interventions

As outlined in Table 4, our preliminary analysis indicated that HMB consumption did not significantly improve serum testosterone concentration. Also, no significant heterogeneity was observed among studies. Subgroup analysis was conducted to check as to how the effect of HMB supplementation on serum testosterone is different according to type of exercise activities (Figure 3) or duration of the interventions (Figure 4). This analysis revealed that the pooled effect of HMB supplementation on testosterone concentration was influenced by the duration of the supplementation (<6 weeks vs. ≥6 weeks), in which a significant increase in testosterone concentration was found in studies that lasted longer than 6 weeks. Also, subgroup analysis based on the type of exercise training indicated that the increasing effect of HMB supplementation on serum testosterone is significant in aerobic and anaerobic combined activities. Subgroup analysis did not show any significant effect of HMB supplementation on testosterone concentration in studies lasting less than six weeks or on resistance training exercise.

**TABLE 4:** Effect of HMB supplementation on testosterone concentration in athletes, subgroup analyses based on study duration and type of exercise activities (all analyses were conducted using random effects model)

Study group	No. of effect sizes	Meta‐analysis		Heterogeneity			
		Effect Mean difference (95%CI)	*P* effect	*Q* statistic	*P* within group	*I^2^* (%)	*P* between group
Duration							
>6 weeks	3	1.60 (0.08, 3.12)	**0.039**	0.28	0.870	0.00	0.188
≤6 weeks	5	0.38 (−0.62, 1.38)	0.455	1.32	0.858	0.00	
Type of exercise activities							
Aerobic and anaerobic combined	4	1.56 (0.07, 3.05)	**0.040**	0.34	0.952	0.00	0.196
Resistance training	4	0.38 (−0.63, 1.38)	0.464	1.32	0.725	0.00	
Overall	8	0.75 (−0.09, 1.58)	0.079	3.33	0.853	0.00	–

Bold values provide significancy *p < .05.

#### 3.3.3 Sensitivity analysis and publication bias

Sensitivity analysis showed that the omission of any of the studies from the meta‐analysis creates no change in the outcomes of the meta‐analysis on testosterone concentration, whereas the results on cortisol concentration were sensitive to omitting 3 studies (Durkalec‐Michalski & Jeszka, 2015; Hoffman et al., 2004; Portal et al., 2011). Funnel plots for cortisol and testosterone were visually symmetrical, and the results of Egger's test did not determine any evidence of publication bias in studies that examine the effect of HMB consumption on cortisol (Egger's test, *p* = .337) and on testosterone (Egger's test, *p* = .140).

##### NutriGrade

Overall scores of quality of the findings, which was evaluated using the NutriGrade scoring system, were 6.3 for cortisol concentration (showing moderate confidence in the effect estimate, which indicates future well‐designed RCTs are still needed to confirm our outcomes), and 4.8 for testosterone concentration (showing low confidence in the effect estimate, which indicates further investigation will provide important evidence on the confidence and likely change the effect estimate).

## 4 DISCUSSION

The efficacy of HMB has not been distinctly established regarding hormonal responses; thus, this systematic review and meta‐analysis provided comprehension into the potential benefits conferred by HMB supplementation to help athletes to make aware decisions on its usage and impact. In this systematic review and meta‐analysis of 7 studies (235 participants), we found that HMB consumption led to a significant decrease in cortisol and increase in testosterone concentrations after resistance exercises and aerobic and anaerobic combined activities, respectively. Several probable mechanisms have been suggested for the beneficial effects of HMB supplementation on testosterone and cortisol concentration in athletes.

Our results support those of past investigations showing that amino acid consumption may play a role in reducing exercise‐induced increases in cortisol (Sharp & Pearson, 2010). Cortisol, is a catabolic hormone and basic glucocorticoid form in humans, secreted from the adrenal cortex in response to psychological and physical stress (Brownlee et al., 2005). During cortisol elevations in exercise, impacts of this hormone happen after exercise within the early recovery. Elevation in serum cortisol concentration, within the physiological range in the short term, motivates proteolysis and augments serum leucine utilization and concentration (Tinsley et al., 2018). The study of Knitter (Knitter et al., 2000) showed that HMB operated as an anticatabolic agent rather than an anabolic agent. The mechanism for this reaction is not exactly obvious. Some studies support the anticatabolic hypothesis of HMB expressed by reductions in the muscle damage indices such as creatine phosphokinase and lactate dehydrogenase (Nissen & Abumrad, 1997; Rahimi et al., 2018) and subsequent lower cortisol levels. The major characteristics of skeletal muscle damage without cell necrosis is the muscle fiber disruption, exclusively the basal lamina sheath. Regarding mechanical stimuli, especially in resistance exercise, it is known that it can boost micro damage in muscle fibers imposed by contractions or overload and depending on the volume, duration, and intensity of training, the degree and severity of damage, and discomfort may be combined over time (Jäger et al., 2019; Nakhostin‐Roohi et al., 2016). As such, HMB can affect muscle damage and cortisol levels more efficiently after resistance exercise. However, more studies should be conducted to reveal physiological and molecular mechanisms by which muscle damage markers were decreased after HMB supplementation.

Moreover, cortisol shows an anticipatory reaction to exercise and physical activity (Suay et al., 1999), and Kraemer et al. previously suggested that this reaction was reduced using a herbal supplement with specifically designed for aerobic exercise (Kraemer et al., 2005). Because amino acid supplementation increases the circulating amino acid concentrations (Matsumoto et al., 2007), possibly less cortisol was needed for gluconeogenesis before, during, and after exercise to break down proteins into amino acids.

Reduced cortisol at rest enhances the anabolic‐to‐catabolic hormone ratio, that in theory, would increase testosterone chronically and muscle tissue protein balance (Kraemer et al., 2009). Also, the main metabolic pathway of HMB is a conversion to HMG‐CoA in the cytosol of liver and muscle, thus HMB is a precursor of cholesterol (Rabinowitz, 1955; Rudney, 1957) and HMB supplementation leads to an increase in synthesis of cholesterol and may act as a structural component of cell membranes (Nissen et al., 1985). Given that damaged muscle cells after exercise (Evans & Cannon, 1991) may lose their ability to make sufficient HMG‐CoA for the synthesis of cholesterol, HMB supplement may be a suitable source of HMG‐CoA to synthesize testosterone (Holecek et al., 2009; Nissen & Abumrad, 1997), however, no study has confirmed this hypothesis and there are no specific data to be able to formulate dose and duration for HMB supplementation. The increment in testosterone concentration seems to be linked with an increase in the endogenous cholesterol that is a substrate in the steroidogenesis process. Additionally, our results in regard to the HMB and aerobic and anaerobic exercise effects on changes in the testosterone response propose that acute ingestion does not increase the testosterone response to exercise. Townsend et al. study carried out 3 exercise protocols throughout the 12 weeks to assay the supplementation acute response. In weeks 6 and 9 of supplementation, the response of testosterone was increased in the intervention group (Townsend et al., 2015). Therefore, chronic supplementation of HMB may lead to a more pronounced response of testosterone. The increase in testosterone concentration could be considered as a beneficial result and demonstrate a better anabolic status in athletes, which plays a key role in training adaptation and recovery in well‐trained athletes (Koundourakis & Margioris, 2019).

To the best of our knowledge, this study is the first systematic review and meta‐analysis that attempts to summarize the role of HMB supplementation on hormonal changes (cortisol and testosterone) in athletes. All included studies administered the same doses of HMB for intervention and all participants were male (except in one study (Portal et al., 2011)) that resulted in a decrement in heterogeneity. Moreover, the absence of heterogeneity among the included studies enhanced the power of our outcomes. However, some limitations should be mentioned. Considering the above items, it is not possible to evaluate the effect of the HMB consumption in female athletes and dose–response relationship between HMB supplementation and hormonal changes. Lack of information about data on frequency and intensity of exercise, genetic background, lifestyle factors, and lack of complete baseline cortisol and testosterone data for subgroup analysis make overall interpretation of the results difficult. Eventually, the type of exercise was varied among the included studies, which could affect the reported outcomes.

In conclusion, the current systematic review and meta‐analysis outcomes revealed that HMB supplementation may be linked with a decrease in cortisol concentration after resistance exercise. In contrast to cortisol, testosterone concentration significantly increased in aerobic and anaerobic combined training. More investigations are recommended with a variety of doses and exercises, longer time in periods and in both sexes in athletes.

## CONFLICT OF INTEREST

None.

## Data Availability

The data that support the findings of this study are available from the corresponding author upon reasonable request.
